# The determinants and impacts of age-disparate relationships on women in Zimbabwe: A life course perspective

**DOI:** 10.1016/j.ssmph.2021.100947

**Published:** 2021-10-21

**Authors:** Angela Y. Chang, Rufurwokuda Maswera, Louisa R. Moorhouse, Morten Skovdal, Constance Nyamukapa, Simon Gregson

**Affiliations:** a Danish Institute for Advanced Study, University of Southern Denmark, Odense, Denmark; b Department of Clinical Research, University of Southern Denmark, Odense, Denmark; c Biomedical Research and Training Institute, Harare, Zimbabwe; d MRC Centre for Global Infectious Disease Analysis, Department of Infectious Disease Epidemiology, School of Public Health, Imperial College London, London, United Kingdom; e Department of Public Health, University of Copenhagen, Copenhagen, Denmark

**Keywords:** Age-disparate relationship, Age disparity, Zimbabwe

## Abstract

Age-disparate relationships (ADR) with older men have been studied mostly in the context of HIV and found to be associated with increased HIV prevalence among young women in sub-Saharan Africa. Less is known about the impact of ADR on the broader life course of women. The objectives of this study are to identify the factors associated with being in ADR and estimate the association between ADR and a set of life outcomes in Manicaland, Zimbabwe. We used data from a general population open-cohort survey from 1998 to 2013 in Manicaland. We applied binary logistic regression models to estimate the odds ratios for association between socio-demographic determinants and ADR and multinomial logistic regression models to estimate the association between ADR and women's life outcomes. We found that women with less education, younger age at first sex and first marriage were more likely to be in ADR, and women in ADR have male partners with less education and less skilled employment. In terms of life and relationship outcomes, women in ADR had mostly negative life outcomes compared to women not in ADR. Future policies and research on ADR in women should reflect these complexities and study a wider range of life outcomes, beyond the commonly studied narrower topics such as HIV.

## Highlights

• Long-term age-disparate relationships (ADR) is a common and long-standing phenomenon in southern Africa.
• In Manicaland, eastern Zimbabwe, we found women with less education, younger age at first sex and first marriage were more likely to be in ADR.
• We found mostly negative (employment, health, etc) but some positive relationships (higher contraceptive use, less unwanted pregnancies) between ADR and women's life outcomes.
• For policymakers and researchers aiming to improve overall wellbeing of women and girls, this paper provides a deeper understanding the reasons why women enter ADR and how these relationships may impact their life outcomes beyond commonly studied HIV and STI transmission.

## 1 Introduction

Age-disparate relationships (ADR), commonly defined as those between younger women and men who are at least five years older, occur in many societies (Lawson et al., 2021). In sub-Saharan Africa, research on ADR has mostly studied the acquisition of HIV and sexually transmitted diseases (STI), and found HIV risk to be higher in women in ADR than in women who were not in ADR (Mwinnyaa et al., 2018; Schaefer et al., 2017). With this evidence, researchers have called for interventions to reduce the formation of ADR in these settings (Beauclair & Delva, 2013; Groves et al., 2020). However, beyond HIV and STI, much less is known about whether ADR is beneficial or harmful to the broader life course of women.

This study was motivated, in part, by Elizabeth Pisani's work on the importance of acknowledging people's own agencies in determining their best choices given the external constraints (Pisani, 2010a, 2010b). Public health researchers and practitioners sometimes hold paternalistic views on certain behaviours, such as drug use, transactional sex, or ADR, and question individuals' rationale for adopting them (Lawson et al., 2021). However, understanding individual agencies and the drivers that lead to these “negative” behaviours is important, not solely for the purpose of designing public health interventions for HIV prevention, but more fundamentally, to respect the study subjects as fellow human beings and learn from their decision-making processes (Pisani 2010a). In the case of ADR, women may not necessarily be passive victims and may hold a degree of agency and, in the context of their social and economic circumstances, make choices that they deem beneficial (Deane and Wamoyia, 2015).

ADR has been a common and longstanding phenomenon in many parts of the world, including settings that are vastly different from those in sub-Saharan African or in other poor environments – think young actresses in Hollywood or high school girls in Tokyo (Elliott 2015; Fifield 2017; Mazurek 2018); therefore, it is reasonable to hypothesize that ADR could, in fact, be beneficial in some dimensions of one's life. The reasons behind this phenomenon and their most common consequences are likely to vary considerably according to the local context but rarely have been described.

In both casual and long-term ADR in sub-Saharan African settings - including in our own study areas in Manicaland, east Zimbabwe - qualitative studies have found that material and financial gains can be the main motivating factor and that girls in these relationships had positive perceptions and attitudes towards ADR (Beauclair & Delva, 2013; De Wet, Alex-Ojei, and Akinyemi 2019; Leclerc-Madlala 2008), despite also associating it with the danger of contracting STI or HIV (Gregson et al., 2002; Leclerc-Madlala 2008; Nkosana 2006). This study focuses specifically on long-term non-casual ADR between younger women and much older men, which are common in Manicaland and other parts of sub-Saharan Africa (Ibisomi 2014; Maughan-Brown, Evans, and George 2016; Schaefer et al., 2017). Existing studies have found mostly negative outcomes: for example, being in long-term ADR was associated with inconsistent and lower contraceptive use in Ethiopia, Nigeria, and South Africa (George et al., 2019; Ibisomi 2014; Kitila et al., 2020), lower psychological wellbeing in Ethiopia but not Niger (John, Edmeades, and Murithi 2019), and less involvement in family decisions and on choice of antenatal care provider in Nigeria (Wright et al., 2020). The only two studies reporting positive outcomes have been those by Adebowale (2018), who found that spousal age-difference was negatively associated with the proportion of women who faced intimate partner violence in Nigeria; and Lawson et al. (2021), who found better mental health state and higher autonomy in household decision-making among women in ADR in northern Tanzania. The population in Manicaland is similar to those studied by Adebowale and Lawson et al. in that bridewealth is practised widely and, for men, having a younger wife may be seen as a means to exercise greater power over the family. As Adebowale notes for Nigeria, Zimbabwe's economy has been in a poor state for several years which has contributed to a situation wherein men frequently postpone marriage until their late 20s or early 30s when they are more likely to have the necessary resources. However, our study setting is more agrarian and has higher levels of wage labour than the Tanzanian setting studied by Lawson et al.

In Manicaland, qualitative studies have found that young women are interested in marrying older men primarily because they are more likely to be able to provide socioeconomic security and status through their having more established and possibly higher-level employment and incomes (Gregson et al., 2002). Other underlying factors contributing to high levels of long-terms ADRs may include the traditional practice of polygyny and, in rural areas, the high female-to-male sex ratios that result largely from higher levels of male labour migration. However, the determinants and outcomes over the life course as a consequence of ADR have not been investigated using quantitative data. The objectives of this study are therefore to identify the factors associated with being in long-term ADR and to estimate the association between ADR and a set of life outcomes in Manicaland, Zimbabwe. More specifically, we test the hypothesis that long-term, non-casual relationships between younger women and much older men could in fact be beneficial for some dimensions of the women's lives.

## 2 Methods

### 2.1 Data

The Manicaland Project was established in 1998 in three districts (Mutasa, Makoni, Nyanga) in Manicaland province, located in eastern Zimbabwe, covering demographic and socioeconomic characteristics, sexual behaviour, HIV and AIDS related knowledge, and uptake of HIV services (Gregson et al., 2017). The project currently has seven completed rounds of a general population open-cohort survey plus an on-going eighth round at the time of writing. This study uses data from the individual interviews in the first six rounds, completed in 2013 (the seventh round was not completed at the time of this study). Participants were selected from a household census in 12 sites (eight in the sixth survey round), representing small towns, farming estates, roadside trading centres, and subsistence farming villages. The main eligibility criteria were being aged 15–54 years and having been resident in a household in one of the study sites for at least 4 nights in the month preceding the initial survey visit. Up to two return visits were made to households where eligible individuals were temporarily absent or otherwise unavailable for interview.

Each survey included between 6000 and 14,000 participants, and participation rates ranged from 73.0% to 79.5%. Individual interviews were conducted using paper questionnaires in survey rounds 1–5, and smartphones and EpiCollect software in round 6. The survey enumerators requested privacy for individual interviews, but sometimes others (mainly children) were presented. Informal confidential voting interview methods were used to reduce social desirability bias in data on sexual behaviour. Most non-participation was due to temporary absences from the household due to high population mobility. Ethical approval for the project was obtained from the Imperial College London Research Ethics Committee and the Medical Research Council of Zimbabwe. Detailed description of the project and survey outcomes can be found elsewhere (Gregson et al., 2017). Previous studies from the project found that HIV prevalence fell from 25.1% at baseline to 15.8% in 2013 (Schaefer et al., 2017).

We created two individual-level datasets, each taking a different subset of the larger dataset. First, we included data from individuals who are women and ever been married or in a long-term relationship (defined as relationships lasting more than one year). Second, to understand more recent trends, we restricted the sample to women aged 30 or younger and who were ever married or in a long-term relationship. For both subsets, we include data from the individual's most recent survey round. Only women who reported their age and their partner's age at the time of marriage were included in the analysis. Among participants with missing information on non-time-varying variables, such as year or birth and age at first sex, we carefully examined if they provided answers in other survey rounds and assigned the most common and logical answer to the missing data point.

### 2.2 Measures

To define ADR, we first estimated both the woman's and her partner's age at the beginning of the relationship, calculated the age difference (in years) and categorized the relationship into less than 5 years, 5–9 years, and 10+ years of age difference. Relationships in which women were older than men were excluded.

**Fig. 1:**
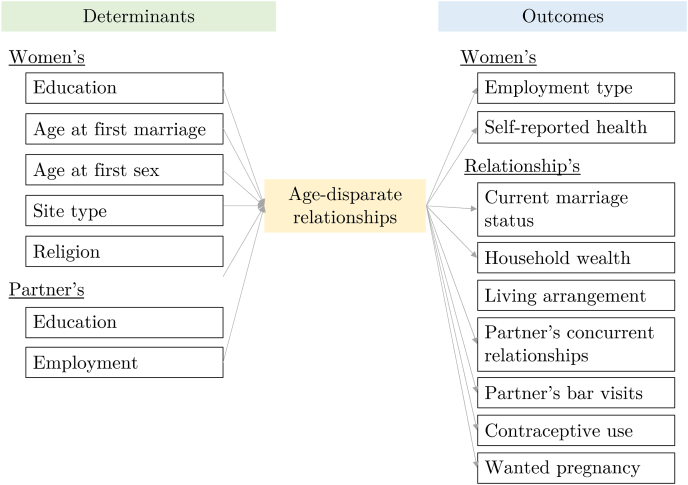
Conceptual framework on the determinants and outcomes of age-disparate relationships.

Fig. 1 depicts the conceptual framework, developed based on existing literature and our data availability, to understand the relationship between the determinants and outcomes of ADR. On the left of Fig. 1, based on the literature and data availability, we listed potential determinants of entering ADR, including women's age at marriage, highest educational level, age at first sex, and partner's age at marriage, highest educational level, and employment type. Highest level of education was defined as none, primary (Level 1, 1–7 years), secondary (Level 2, 1–6 years), and higher (Level 3, 1–6 years). Categories of employment type included unemployed, informal work (such as petty trading, substance agriculture) and manual/unskilled labour, self-employed, skilled labour, and professional or managerial work. On the right of Fig. 1, we listed potential life outcomes associated with being in ADR, including women's outcomes, such as her employment type and self-reported recent health, and the quality of the relationship, including current marriage status (still in union, separated, divorced, widowed), household wealth, living arrangement, number of partner's concurrent relationships, frequency of the partner's visits to bars or beer halls, frequency of contraception use, and whether the most recent pregnancy was wanted or unwanted. Self-reported health was recorded as being in good health, recurring sickness, or serious illness in the last few months. Household wealth quintile was calculated based on asset ownership, and it refers to the household of the women at the time of the interview (i.e., the household post-relationship, not the original household in which she spent her childhood) (Schur et al., 2015). Living arrangements were described as living together all the time, living together but occasionally apart for work reasons, living together but separated for a period every year for work reasons, living apart but regular/frequent cohabitation, and living apart with infrequent cohabitation. Women were asked whether her partner visited a bar or beerhall in the last month. Frequency of contraception use was reported as none, some of the time, or most/all the time in the last two or three years. Women who ever gave birth were asked whether for their most recent pregnancy they wanted to become pregnant at that time, wanted to wait until later, or not want to become pregnant at all. More details can be found elsewhere (Gregson et al., 2017).

### 2.3 Statistical analysis

**Fig. 2:**
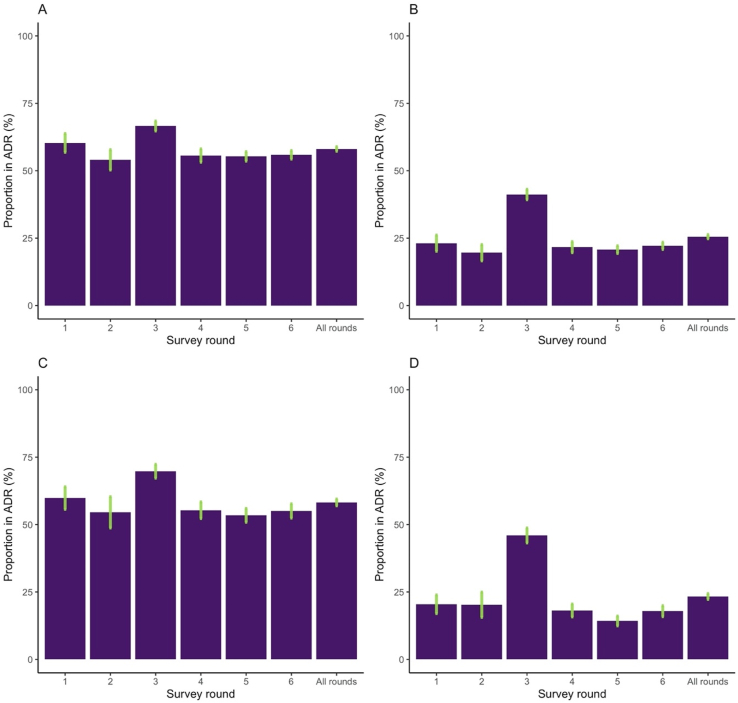
Trend of proportion of age-disparate relationships over timePanel. A: women of all ages, ADR defined as age difference of five or more years. Panel B: women of all ages, ADR defined as age difference of ten or more years. Panel C: women younger than age 30 at the time of survey, ADR defined as age difference of five or more years. Panel D: women younger than age 30 at the time of survey, ADR defined as age difference of ten or more years.

The relationship between each potential determinant and ADR was tested with two logistic regression models. The first model treated ADR as a binary dependent variable and included women's age and survey round (to control for unmeasured temporal confounders) as covariates. The second model further included two other covariates, women's highest educational level and household wealth quintile (as supported by existing literature (add cite)) as covariates. To build Model 2, we tested models with different combinations of the four covariates and used AIC model selection to select the right model (linear with no interaction terms). The relationships between ADR and life outcomes were tested with multinomial logistic regression models, with ADR as a binary independent variable and with the same two sets of covariates as described above. We estimated the odds ratios (OR) and 95% confidence intervals (CI) comparing women in ADR and women not in ADR. We conducted the analyses using the full dataset including women of all ages, as well as the dataset including only women younger than 30 years at the time of the survey. In the main manuscript, we focus on ADR defined as age differences of five or more years and present the full results with ADR defined as ten or more years and women younger than age 30 in the Appendix (Fig. 1, Fig. 2).

Three sets of sensitivity analyses were conducted. First, we removed data from survey round 3 (see reason below) to check for robustness. Second, among individuals without ADR data (Table 1), we randomly assigned them to one ADR status and conducted the same sets of analyses. Third, we only included women who were still married or in union at the time of the survey.

**Table 1:** Socio-demographic characteristics of the study population.

	All ages			Ages≤30		
	In ADR	Not in ADR	ADR data missing	In ADR	Not in ADR	ADR data missing
n	7108	5131	4403	3389	2431	1799
In ADR with >=10-year difference in age (%)	3126 (44.0)	0 (0.0)	NA	1358 (40.1)	0 (0.0)	NA
Survey round (%)						
1 (1998–2000)	459 (6.5)	302 (5.9)	403 (9.2)	316 (9.3)	212 (8.7)	212 (11.8)
2 (2001–2003)	361 (5.1)	307 (6.0)	229 (5.2)	156 (4.6)	130 (5.3)	59 (3.3)
3 (2003–2005)	1657 (23.3)	831 (16.2)	1827 (41.5)	859 (25.3)	372 (15.3)	929 (51.6)
4 (2006–2008)	863 (12.1)	688 (13.4)	454 (10.3)	549 (16.2)	443 (18.2)	217 (12.1)
5 (2009–2011)	1677 (23.6)	1354 (26.4)	718 (16.3)	764 (22.5)	666 (27.4)	208 (11.6)
6 (2012–2013)	2091 (29.4)	1649 (32.1)	772 (17.5)	745 (22.0)	608 (25.0)	174 (9.7)
Age (mean (SD))	32.8 (11.3)	32.8 (10.4)	35.00 (11.5)	23.5 (3.7)	24.4 (3.4)	24.00 (3.6)
Household wealth index (%)						
1 (Poorest)	852 (12.2)	573 (11.4)	576 (13.4)	486 (14.6)	321 (13.5)	235 (13.5)
2	4186 (59.9)	2958 (58.6)	2542 (59.3)	1877 (56.5)	1339 (56.3)	981 (56.2)
3	1447 (20.7)	1092 (21.6)	879 (20.5)	705 (21.2)	511 (21.5)	396 (22.7)
4	486 (7.0)	408 (8.1)	278 (6.5)	244 (7.3)	198 (8.3)	127 (7.3)
5 (Richest)	17 (0.2)	17 (0.3)	10 (0.2)	8 (0.2)	9 (0.4)	5 (0.3)
Highest education level (%)						
None	257 (3.6)	95 (1.9)	243 (5.5)	35 (1.0)	14 (0.6)	18 (1.0)
Primary	2807 (39.5)	1549 (30.2)	1919 (43.6)	870 (25.7)	446 (18.3)	528 (29.3)
Secondary	3993 (56.2)	3402 (66.3)	2208 (50.1)	2463 (72.7)	1930 (79.4)	1245 (69.2)
Higher	51 (0.7)	85 (1.7)	33 (0.7)	21 (0.6)	41 (1.7)	8 (0.4)
Current marriage status (%)						
Still in union	6163 (86.7)	4580 (89.3)	1413 (32.1)	3153 (93.0)	2302 (94.7)	786 (43.7)
Divorced or separated	386 (5.4)	248 (4.8)	1628 (37.0)	177 (5.2)	101 (4.2)	841 (46.7)
Widowed	559 (7.9)	303 (5.9)	1361 (30.9)	59 (1.7)	28 (1.2)	172 (9.6)
Site type (%)						
Subsistence farming	2452 (34.5)	1754 (34.2)	1472 (33.4)	1091 (32.2)	782 (32.2)	581 (32.3)
Agricultural estate	1996 (28.1)	1368 (26.7)	1261 (28.6)	1026 (30.3)	678 (27.9)	537 (29.8)
Migrated	34 (0.5)	20 (0.4)	11 (0.2)	25 (0.7)	10 (0.4)	8 (0.4)
Roadside settlement	1362 (19.2)	1031 (20.1)	823 (18.7)	563 (16.6)	481 (19.8)	302 (16.8)
Town	1264 (17.8)	958 (18.7)	836 (19.0)	684 (20.2)	480 (19.7)	371 (20.6)
Church denomination (%)						
Christians	3554 (55.1)	2648 (56.6)	2242 (55.9)	1538 (50.8)	1218 (55.3)	852 (52.9)
Spiritualists	1272 (19.7)	950 (20.3)	615 (15.3)	618 (20.4)	470 (21.4)	220 (13.7)
Traditionalists	73 (1.1)	31 (0.7)	70 (1.7)	39 (1.3)	11 (0.5)	27 (1.7)
None	276 (4.3)	157 (3.4)	236 (5.9)	165 (5.5)	88 (4.0)	121 (7.5)
Other	1270 (19.7)	890 (19.0)	846 (21.1)	667 (22.0)	414 (18.8)	391 (24.3)
Employment sector and type (%)						
Professional/managerial	103 (1.4)	153 (3.0)	62 (1.4)	37 (1.1)	48 (2.0)	12 (0.7)
Self-employed	3 (0.3)	26 (0.5)	15 (0.3)	11 (0.3)	10 (0.4)	7 (0.4)
Skilled labour	121 (1.7)	116 (2.3)	130 (3.0)	47 (1.4)	39 (1.6)	40 (2.2)
Informal/unskilled	1607 (22.6)	1111 (21.7)	1437 (32.6)	715 (21.1)	498 (20.5)	538 (29.9)
Unemployed	5185 (72.9)	3679 (71.7)	2719 (61.8)	2538 (74.9)	1810 (74.5)	1184 (65.8)
Age at first marriage (mean (SD))	18.4 (2.6)	19.7 (2.9)	19.0 (3.1)	18.0 (2.3)	19.2 (2.5)	18.6 (2.5)
Age at first sex (mean (SD))	17.9 (2.5)	19.1 (2.8)	18.4 (2.9)	17.7 (2.2)	18.8 (2.4)	18.1 (2.5)
Self-reported health in the last few months (%)						
Good health	5729 (81.0)	4223 (82.9)	3317 (75.7)	2895 (85.7)	2108 (87.0)	1472 (82.1)
Recurring sickness	1117 (15.8)	751 (14.7)	870 (19.9)	378 (11.2)	275 (11.3)	252 (14.1)
Serious illness	230 (3.3)	123 (2.4)	192 (4.4)	104 (3.1)	40 (1.7)	69 (3.8)
Age of partner at marriage (mean (SD))	29.6 (7.5)	21.5 (3.0)	NA	29.0 (8.0)	21.3 (2.6)	NA
Partner employment sector and type (%)						
Professional/managerial	476 (6.7)	376 (7.3)	16 (0.4)	239 (7.1)	158 (6.5)	5 (0.3)
Self-employed	139 (2.0)	76 (1.5)	1 (0.0)	77 (2.3)	42 (1.7)	0 (0.0)
Skilled labour	1138 (16.0)	807 (15.7)	28 (0.6)	598 (17.6)	390 (16.0)	5 (0.3)
Informal/unskilled	2261 (31.8)	1680 (32.7)	137 (3.1)	1295 (38.2)	894 (36.8)	39 (2.2)
Unemployed	2211 (31.1)	1590 (31.0)	119 (2.7)	977 (28.8)	799 (32.9)	20 (1.1)
Frequency of contraception use in the last 2–3 years (%)						
None	2425 (53.3)	1749 (51.7)	1629 (71.2)	1504 (57.3)	1058 (56.0)	849 (66.3)
Some of the time	442 (9.7)	274 (8.1)	316 (13.8)	322 (12.3)	195 (10.3)	247 (19.3)
Most/all of the time	1685 (37.0)	1363 (40.3)	344 (15.0)	798 (30.4)	635 (33.6)	184 (14.4)
Whether recent pregnancy desired (%)						
Yes	2581 (49.4)	1687 (45.8)	1994 (60.7)	1362 (46.8)	884 (43.9)	810 (51.3)
Later	481 (9.2)	380 (10.3)	244 (7.4)	314 (10.8)	250 (12.4)	163 (10.3)
Not at all	2161 (41.4)	1613 (43.8)	1049 (31.9)	1236 (42.4)	880 (43.7)	607 (38.4)
Partner visited a bar or beer-hall in the last month (%)	2654 (43.5)	1835 (41.4)	92 (31.7)	1338 (43.0)	870 (38.9)	26 (40.6)
Partner's highest education level (%)						
None	132 (2.1)	32 (0.7)	21 (7.1)	39 (1.2)	6 (0.3)	2 (2.9)
Primary	2035 (32.6)	1070 (23.4)	102 (34.5)	754 (23.6)	365 (15.8)	33 (47.8)
Secondary	3728 (59.7)	3193 (69.8)	146 (49.3)	2189 (68.6)	1772 (76.7)	21 (30.4)
Higher	346 (5.5)	279 (6.1)	27 (9.1)	211 (6.6)	167 (7.2)	13 (18.8)
Living arrangement with partner (%)						
Living together all times	3782 (60.7)	2694 (59.0)	192 (65.3)	1843 (57.8)	1332 (57.7)	41 (60.3)
Living together with occasional trips	832 (13.4)	650 (14.2)	28 (9.5)	509 (16.0)	372 (16.1)	12 (17.6)
Living away for a period (seasonal)	337 (5.4)	276 (6.0)	9 (3.1)	176 (5.5)	126 (5.5)	4 (5.9)
Living apart with regular visits	1104 (17.7)	789 (17.3)	53 (18.0)	584 (18.3)	403 (17.5)	9 (13.2)
Living apart	171 (2.7)	158 (3.5)	12 (4.1)	79 (2.5)	76 (3.3)	2 (2.9)

All analyses were done using R, version 4.0.1.

## 3 Results

A total of 16,642 women across all ages met the inclusion criteria, of which 7619 were younger than age 30 (Table 1). Including only women who reported their age and their partner's age at the time of marriage resulted in a total of 12,239 (74%) women of all ages and 5280 (69%) women younger than 30 years old. Mean age in both ADR and non-ADR groups were 32.8 years. Comparing individuals without data on ADR to those with data, they were slightly older (mean age 35.0), proportionally more were in survey round 3, had lower levels of education, and more were divorced, separated, or widowed.

Across all age groups and survey rounds, 58.1% [95% CI 57.2–59.0] and 25.5% [24.8–26.3] were ever in ADR with age differences of 5+ and 10+ years, respectively (Fig. 2, panels A and B). The proportion of women in relationships with five or more years of age difference was 60.3% [56.8–63.8], 54.0% [50.3–57.8], 66.6% [64.7–68.5], 55.6% [53.6–58.1], 55.3% [53.6–57.1], and 55.9% [54.3–57.5] across the six survey rounds, respectively. The proportion in relationships with ten or more years of age difference was 23.1% [20.1–26.1], 19.6% [16.6–22.6], 41.2% [39.3–43.1], 21.7% [19.6–23.7], 20.8% [19.3–22.2], and 22.2% [20.8–23.5] across the six survey rounds, respectively. The differences across rounds are not statistically significant except for the higher peaks in round 3. We therefore conducted sensitivity analysis by removing data from round 3 to test for robustness of the results. Among women younger than age 30 at the time of the survey, 58.2% [57.0–59.5] and 23.3% [22.2–24.4] were ever in ADR with age differences of 5+ and 10+ year age differences, respectively (Fig. 2, panels C and D). Similar to the full sample, the proportions do not change widely over time.

### 3.1 Factors associated with being in ADR

**Fig. 3:**
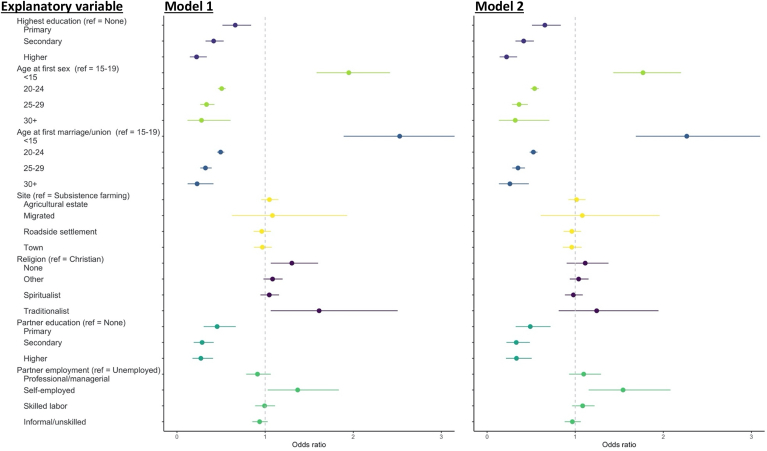
Association between determinants and entering age-disparate relationships (ADR). Model 1 includes women's age and survey round as covariates; model 2 includes women's age, survey round, women's highest educational level, and household wealth as covariates. The reference level of each model is listed in the parenthesis (“ref”). The vertical dash line represents odds ratio at 1. Odds ratios and 95% confidence intervals were calculated comparing women with ADR to women not in ADR. Sample size for each analysis: education 12039; age at first sex 12015; age at first marriage 12039; site 12239; religion 11121; partner's education 10167; partner employment 10532. Sample sizes for each analysis differ due to missing data.

We focus on reporting the results from Model 2, which controlled for age, round, education, and wealth quintile (Model 2 in Fig. 3). Clear gradients were found in women's highest education level, age at first sex, age at first marriage, and the partners' highest education level. Women with lower educational level were more likely to be in ADR, those with primary, secondary, and higher education had 34.4% [OR 0.66, 95% CI 0.51–0.84], 58.5% [OR 0.42, 95% CI 0.32–0.53], and 77.9% [OR 0.22, 95% CI 0.14–0.34] lower odds of being in ADR than women with no education. Higher age at first sex was associated with lower odds: age at first sex of 20–24, 25–29, and 30+ had 46.0% [OR 0.54, 95% CI 0.49–0.59], 63.6% [OR 0.36, 95% CI 0.28–0.46], and 68.0% [OR 0.32, 95% CI 0.14–0.71] lower odds compared to women who first had sex at ages 15–19. In comparison, women who had sex younger than age 15 had much higher odds of being in ADR (OR 1.77, 95% CI 1.43–2.20). Older age at first marriage was also associated with lower odds and follow similar patters as age at first sex. Site type and religion were not associated with women being in ADR of five or more years. However, those who did not belong to any religion had higher odds of being in ADR [OR 1.34, 95% CI 1.07–1.66) of 10+ years age difference than Christians (the reference group), and younger women belonging to the Traditionalist religions were more likely to be in ADR [OR 2.08, 95% CI 1.08–4.34] (Table A1; Figure A1). On partners' characteristics, women in ADR were more likely to have partners with less education: partners had 50.9% [OR 0.49, 95% CI 0.32–0.72], 66.9% [OR 0.33, 95% CI 0.22–0.49], and 66.6% [OR 0.33, 95% CI 0.21–0.51] lower odds of being in primary, secondary, and higher education, compared to having no education. Partners of women in ADR were more likely to be self-employed than unemployed but no significant differences were found in other employment types. Across all results, the results were qualitatively similar for most variables with the higher ADR threshold of 10-year age difference.

### 3.2 Association between ADR and life outcomes

**Fig. 4:**
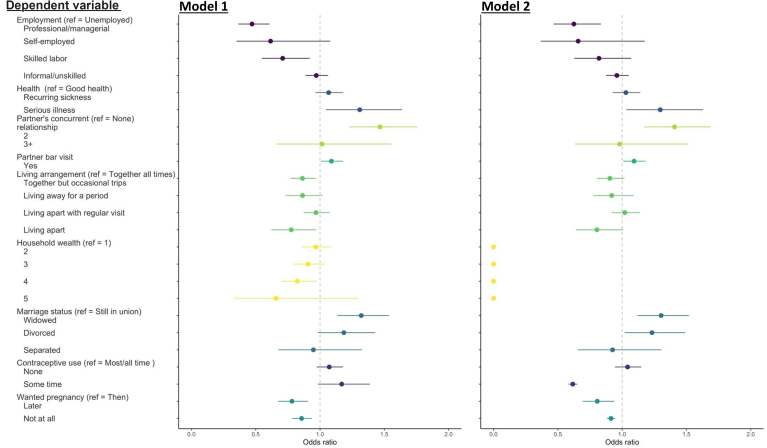
Association between age-disparate relationships (ADR) and life and relationship outcomes. Model 1 includes women's age and survey round as covariates; model 2 includes women's age, survey round, women's highest educational level, and household wealth as covariates. The reference level of each model is listed in the parenthesis (“ref”). The vertical dash line represents odds ratio at 1. Odds ratios and 95% confidence intervals were calculated comparing women with ADR to women not in ADR. Sample size for each analysis: employment 11922; health status 11973; concurrent relationship 7148; bar visits 10345; living arrangement 10595; household wealth 12039; marriage status 12038; contraceptive use 7808; pregnancy 8760. Sample sizes for each analysis differ due to missing data.

Being in ADR was associated with lower odds of women being employed and in higher-skill jobs compared to women not in ADR (Fig. 4). For example, women in ADR were 37.5% [OR 0.62, 95% CI 0.47–0.83] less likely to be in professional or managerial roles. Being in ADR was also associated with 29.7% [OR 1.30, 95% CI 1.03–1.63] higher odds of having experienced serious illness in the last few months. Restricting to ADR with more than 10-year age difference, being in ADR was associated with 16.5% [OR 1.17, 95% CI 1.04–1.3] and 57.7% [OR 1.58, 95% CI 1.26–1.98] higher odds of having recurring or serious illnesses, respectively (Figure A2). Among women younger than age 30, the odds of having a serious illness was 89.7% [OR 1.90, 95% CI 1.30–2.78] higher (Table A2; Figure A2). On partner and relationship characteristics, partners of women in ADR had higher odds of having another concurrent partner (OR 1.41 [1.17–1.69]) and visited bar or beerhall (OR 1.09 [1.01–1.18]) in the last month. Higher household wealth was associated with lower but non-statistically significant odds (except for quintile 4) in model 1, but this relationship disappeared after controlling for women's education level. Women in ADR had higher odds of being divorced (OR 1.23 [1.02–1.49)) and widowed (OR 1.30 [1.12–1.52]), the latter likely due to older ages of partners. The association is stronger for both ADR of ten years and among women younger than age 30. Women in ADR of five or more years reported higher frequent use of contraception (lower odds of answering “some time”), while women in ADR of ten or more years reported lower frequent use (higher odds of answering “none” or “some time”). On the other hand, women in ADR (both five- and ten-year differences, and women younger than age 30) had lower odds of having unwanted pregnancy than those not in ADR.

### 3.3 Sensitivity analysis

We focus on reporting the statistically significant changes in the results of Model 2. The full analyses can be found in the Appendix (Tables A1-2). Results on the determinants of ADR were not sensitive to either sets of sensitivity analyses (Table A1). In contrast, we found some results on the relationship between ADR and life outcomes to be sensitive. First, the statistically significant relationship between ADR and women's self-reported health status (women in ADR more likely to report worse health) disappeared across all three sets of sensitivity analyses. Second, after removing data from survey round 3, the relationships between being in ADR and contraception use and wanted pregnancy disappear once survey round 3 is removed, likely due to reduction in sample size.

## 4 Discussion

This paper contributes to the relatively small set of literature that explores the potential factors associated with women entering ADR and the life outcomes associated with being in such a relationship in sub-Saharan Africa. Overall, from assessing the determinants and consequences of ADR as covariates in separate logistic regression models, we find that younger women with lower education and younger age at sexual debut or first marriage are more likely to enter ADR with men with lower socio-economic status, and being in ADR is also associated with mostly negative life outcomes.

We present three key findings. First, there has not been a noticeable change in the proportion of women in relationships with older men between 1998 and 2013. Across all survey rounds, close to 60% were ever in ADR with age differences of greater than 5 years, and more than 25% in ADR with age differences of greater than 10 years. This is in line with the prevalence rates reported by other studies in sub-Saharan Africa (Ibisomi 2014; Maughan-Brown, Evans, and George 2016; Schaefer et al., 2017). Second, women with lower education, younger age at sexual debut or first marriage are at higher odds of being in ADR. We further found that women in ADR were more likely to have partners with lower education or who are unemployed than women not in ADR. Third and the most interesting set of findings is on the relationship between being in ADR and women's life outcomes and the quality of the relationship. Contrary to our hypothesis, across most outcomes, women in ADR had worse life outcomes and poorer quality of their relationship in this setting compared to women not in ADR. They reported worse health outcomes and employment opportunities, more likely to be divorced or widowed, and their partners were more likely to have a concurrent relationship and to visit beerhalls or bars. The relationships between being in ADR and negative outcomes were stronger among women in relationships with age-differences of 10 years or more: these women were less likely to be in professional or managerial roles, more likely to be widowed, divorced, or separated, and more likely to have concurrent partners. The higher likelihood of the partner having a concurrent relationship was previously found to be associated with higher prevalence of HIV among these women (Schaefer et al., 2017). In contrast, one surprising positive finding was that women in ADR were more likely to use contraception and less likely to have unwanted pregnancies, in line with some findings from Beauclair et al. (2016) in Malawi.

Given the high ADR prevalence in this setting, it is possible that culturally long-term ADR is acceptable, and women (and their families) may in fact actively reject marrying their age-peers. While promoting interventions to reduce the formation of ADR may in the short-term reduce HIV transmission (Beauclair & Delva, 2013; Groves et al., 2020), failing to consider the complexity of why these relationships continue to exist (with no clear declining trends in prevalence) and without considering women's own agencies may lead to unintended consequences beyond the immediate health outcomes (Hallett et al., 2007). Without judging women's decisions to enter ADR, our findings call for a two-pronged education approach that enhances their opportunities to make informed decisions about the kind of relationships they form, potentially minimizing the long-term consequences of ADR for women. First, girls need to stay in school for as long as possible, and social protection schemes providing financial and educational support to vulnerable families are critical to this objective (Baird et al., 2013), as well as to improve their HIV outcomes (Cluver et al., 2015). Second, Wamoyi et al. (2018) argue that adolescent girls and young women need to reflect on the perceived short-term benefits of ADR in relation to potential medium- and long-term consequences of ADR. This article provides the much-needed knowledge foundation for such reflection. It can either form part of the sexual and reproductive health education curriculum, or constitute a targeted school-based intervention aiming at establishing safe social spaces (see Santhya and Jejeebhoy 2015) for girls to obtain the networks, skills, and information needed to critically reflect on the potential life course impacts of ADR.

The findings from this study should be viewed in the context of the following limitations. First, and the most important limitation is the concern of reverse causality. For example, the relationship between educational attainment and ADR could run both ways – those with lower education are more likely to enter ADR, or those who enter ADR subsequently drop out of school. The majority of women who got married also drop out of school in the same survey round. Since each survey round covers approximately two years, these major life events frequently occur during the same interview round, making it difficult for us to decipher which came first. Contextually, it is common for female students who get married or pregnant to leave school in Manicaland (Gregson, Waddell, and Chandiwana 2001; Lloyd and Mensch 2008). One limitation in data collection is that women change households and often surnames after marriage, making it difficult for follow up. Women may also marry into the husband's village which may be outside of the study area. This may have led to a selection bias, in which women who remain in the sample have mostly remained or married into rural areas. In other words, our findings are applicable to women who are or previously married into rural Zimbabwe. Second, while previous studies in similar settings have found that women from lower income households are more likely to be in ADR, we were not able to test this association because we only collected household wealth at the time of the survey, which reflects the wealth level of the post-marriage and not pre-marriage household. We found that women in ADR are less likely to be in a higher wealth quintile, but we cannot determine the direction of the relationship (whether being in ADR leads to lower wealth or men in lower wealth quintiles are more likely to go into ADR). Third, reporting bias of the partner's age may have led to underreporting of ADR, although given the high prevalence it may not be discriminated against (Helleringer, Kohler, and Mkandawire 2011). Similarly, self-reported outcomes, such as contraceptive use and desired pregnancy, may be subjected to social desirability bias. In addition, there may be unobserved confounding factors that were not included in the analysis.

## 5 Conclusion

ADR is a common and long-standing phenomenon in many societies. For policymakers and researchers aiming to improve overall wellbeing of women and girls, understanding the reasons why women enter ADR and how these relationships may impact their life outcomes beyond commonly studied HIV and STI transmission is important. We found mixed relationship (mostly negative but some positive associations) between ADR and some life outcomes. For a more comprehensive picture, future research and discussions on ADR should expand their perspectives to incorporate a wider range of life outcomes such as health, employment, education, empowerment, poverty, and violence outcomes.

## Author contributions

AYC conceptualized this study with input from MS and SG. SG, RM, and CN designed the original study through which data was collected and provided study oversight and implementation. LM extracted the dataset from the wider study database. AYC conducted all analyses with input from MS, LM and SG. AYC prepared the manuscript. All authors contributed to interpretation of results and read and approved the final article.

## Funding source

Funding for data collection was supported by the 10.13039/100010269Wellcome Trust (reference: 084401/Z/07/B). SG, CN, and LRM are supported by the 10.13039/100000865Bill and Melinda Gates Foundation (reference: INV-023210). CN, LRM and SG acknowledge funding support from the MRC Centre for Global Infectious Disease Analysis (reference MR/R015600/1), jointly funded by the 10.13039/501100000265UK Medical Research Council (MRC) and the UK Foreign, Commonwealth and Development Office (FCDO), under the MRC/FCDO Concordat agreement and is also part of the EDCTP2 programme supported by the European Union.

## Ethical statement

Ethical approval for the project was obtained from the Imperial College London Research Ethics Committee and the Medical Research Council of Zimbabwe.
