# Supplementary material for: The determinants and impacts of age-disparate relationships on women in Zimbabwe: A life course perspective
Source: SSM Popul Health. 2021 Oct 21;16:100947. doi: 10.1016/j.ssmph.2021.100947 (PMC8590073; doi:10.1016/j.ssmph.2021.100947)
Supplement: Multimedia component 1 [file mmc1.pdf]

The determinants and impacts of age-disparate relationships on women in Zimbabwe: A life course perspective

**Online supplementary materials**

## Table of Contents


1. Full results ........ 2
2. Sensitivity analyses ........ 8

**Full results**

**Results 1: Association between determinants and entering age-disparate relationships**

Table A1. Association between determinants and entering age-disparate relationships

| | Odds ratios (95% confidence interval) | | | | | |
|---|---|---|---|---|---|---|
| | All ages, ADR defined as age difference greater than 5 years | | All ages, ADR defined as age difference greater than 10 years | | Ages <=30, ADR defined as age difference greater than 5 years | |
| | Model 1 | Model 2 | Model 1 | Model 2 | Model 1 | Model 2 |
| Highest education level (ref = None) | | | | | | |
| Primary | 0.66 [0.52, 0.84] | 0.66 [0.51, 0.84] | 0.61 [0.49, 0.76] | 0.6 [0.48, 0.75] | 0.81 [0.41, 1.49] | 0.83 [0.43, 1.54] |
| Secondary | 0.42 [0.32, 0.53] | 0.42 [0.32, 0.53] | 0.38 [0.31, 0.48] | 0.38 [0.3, 0.48] | 0.54 [0.28, 0.99] | 0.56 [0.29, 1.03] |
| Higher | 0.22 [0.15, 0.34] | 0.22 [0.14, 0.34] | 0.13 [0.07, 0.24] | 0.14 [0.07, 0.26] | 0.24 [0.1, 0.53] | 0.24 [0.1, 0.54] |
| Age at first sex (ref = 15-19 years) | | | | | | |
| < 15 | 1.95 [1.58, 2.42] | 1.77 [1.43, 2.2] | 1.75 [1.45, 2.1] | 1.58 [1.31, 1.9] | 1.95 [1.44, 2.69] | 1.8 [1.32, 2.5] |
| 20-24 | 0.51 [0.47, 0.55] | 0.54 [0.49, 0.59] | 0.54 [0.49, 0.6] | 0.59 [0.53, 0.65] | 0.49 [0.43, 0.56] | 0.51 [0.45, 0.58] |
| 25-29 | 0.34 [0.26, 0.43] | 0.36 [0.28, 0.46] | 0.5 [0.37, 0.67] | 0.54 [0.39, 0.74] | 0.34 [0.21, 0.55] | 0.35 [0.21, 0.58] |
| 30+ | 0.28 [0.12, 0.61] | 0.32 [0.14, 0.71] | 0.73 [0.28, 1.67] | 0.84 [0.32, 1.94] | N/A | N/A |
| Age at first marriage (ref = 15-19 years) | | | | | | |
| < 15 | 2.53 [1.89, 3.44] | 2.27 [1.69, 3.1] | 1.83 [1.45, 2.31] | 1.63 [1.28, 2.06] | 2.47 [1.64, 3.85] | 2.31 [1.52, 3.64] |
| 20-24 | 0.49 [0.46, 0.54] | 0.53 [0.48, 0.57] | 0.58 [0.53, 0.64] | 0.64 [0.58, 0.71] | 0.49 [0.43, 0.55] | 0.51 [0.45, 0.58] |
| 25-29 | 0.32 [0.26, 0.39] | 0.35 [0.29, 0.43] | 0.49 [0.38, 0.62] | 0.55 [0.42, 0.7] | 0.28 [0.18, 0.43] | 0.3 [0.19, 0.45] |
| 30+ | 0.23 [0.12, 0.41] | 0.26 [0.14, 0.47] | 0.53 [0.25, 1.03] | 0.61 [0.28, 1.2] | N/A | N/A |
| Site type (ref = Subsistence farming) | | | | | | |
| Agricultural estate | 1.05 [0.96, 1.15] | 1.02 [0.92, 1.12] | 1.11 [1, 1.24] | 1.07 [0.96, 1.19] | 1.08 [0.95, 1.24] | 1.03 [0.9, 1.19] |
| Migrated | 1.08 [0.62, 1.93] | 1.08 [0.61, 1.96] | 0.46 [0.21, 0.91] | 0.58 [0.26, 1.15] | 1.6 [0.78, 3.53] | 1.66 [0.8, 3.71] |

| | | | | | | |
|---|---|---|---|---|---|---|
| Roadside settlement | 0.96 [0.87, 1.07] | 0.96 [0.87, 1.07] | 0.97 [0.86, 1.09] | 0.98 [0.87, 1.1] | 0.86 [0.74, 1.01] | 0.85 [0.73, 1] |
| Town | 0.97 [0.87, 1.08] | 0.96 [0.86, 1.08] | 1.07 [0.95, 1.21] | 1.09 [0.96, 1.24] | 1.05 [0.9, 1.22] | 1.02 [0.87, 1.19] |
| Church denomination (ref = Christians) | | | | | | |
| None | 1.3 [1.06, 1.6] | 1.11 [0.9, 1.38] | 1.63 [1.32, 2.01] | 1.34 [1.07, 1.66] | 1.43 [1.09, 1.88] | 1.21 [0.91, 1.61] |
| Other | 1.08 [0.98, 1.2] | 1.04 [0.94, 1.15] | 1.16 [1.04, 1.3] | 1.11 [0.99, 1.25] | 1.29 [1.12, 1.49] | 1.23 [1.06, 1.43] |
| Spiritualists | 1.05 [0.95, 1.16] | 0.98 [0.88, 1.09] | 1.14 [1.01, 1.28] | 1.07 [0.95, 1.2] | 1.1 [0.95, 1.27] | 1.04 [0.89, 1.21] |
| Traditionalists | 1.61 [1.06, 2.5] | 1.24 [0.81, 1.94] | 1.89 [1.26, 2.81] | 1.39 [0.91, 2.09] | 2.39 [1.25, 4.95] | 2.08 [1.08, 4.34] |
| Partner's highest education level (ref = None) | | | | | | |
| Primary | 0.46 [0.3, 0.67] | 0.49 [0.32, 0.72] | 0.47 [0.34, 0.64] | 0.5 [0.36, 0.69] | 0.32 [0.12, 0.71] | 0.33 [0.12, 0.74] |
| Secondary | 0.29 [0.19, 0.42] | 0.33 [0.22, 0.49] | 0.21 [0.16, 0.29] | 0.24 [0.18, 0.34] | 0.2 [0.08, 0.45] | 0.22 [0.08, 0.49] |
| Higher | 0.27 [0.18, 0.41] | 0.33 [0.21, 0.51] | 0.16 [0.11, 0.23] | 0.19 [0.13, 0.28] | 0.18 [0.07, 0.41] | 0.21 [0.08, 0.47] |
| Partner employment sector and type (ref = Unemployed) | | | | | | |
| Professional/managerial | 0.91 [0.79, 1.06] | 1.1 [0.93, 1.29] | 0.67 [0.55, 0.8] | 0.91 [0.74, 1.11] | 1.25 [1, 1.57] | 1.45 [1.14, 1.85] |
| Self-employed | 1.37 [1.03, 1.84] | 1.54 [1.15, 2.08] | 0.82 [0.58, 1.13] | 1.03 [0.72, 1.43] | 1.57 [1.07, 2.34] | 1.78 [1.2, 2.69] |
| Skilled labor | 0.99 [0.89, 1.11] | 1.09 [0.97, 1.22] | 0.84 [0.74, 0.95] | 1 [0.87, 1.14] | 1.22 [1.04, 1.43] | 1.26 [1.07, 1.49] |
| Informal/unskilled | 0.94 [0.86, 1.03] | 0.97 [0.88, 1.06] | 0.84 [0.76, 0.94] | 0.89 [0.8, 0.99] | 1.13 [0.99, 1.29] | 1.14 [1, 1.3] |

Figure A1. Association between determinants and entering age-disparate relationships

Panel A: same as presented in the main manuscript (Figure 3), in which ADR is defined as age difference of five or more years.

Panel B: restricting ADR to age difference of ten or more years.

Panel C: restricting to women ages 30 and younger.

Model 1 includes women's age and survey round as covariates

Model 2 includes women's age, survey round, women's highest educational level, and household wealth as covariates

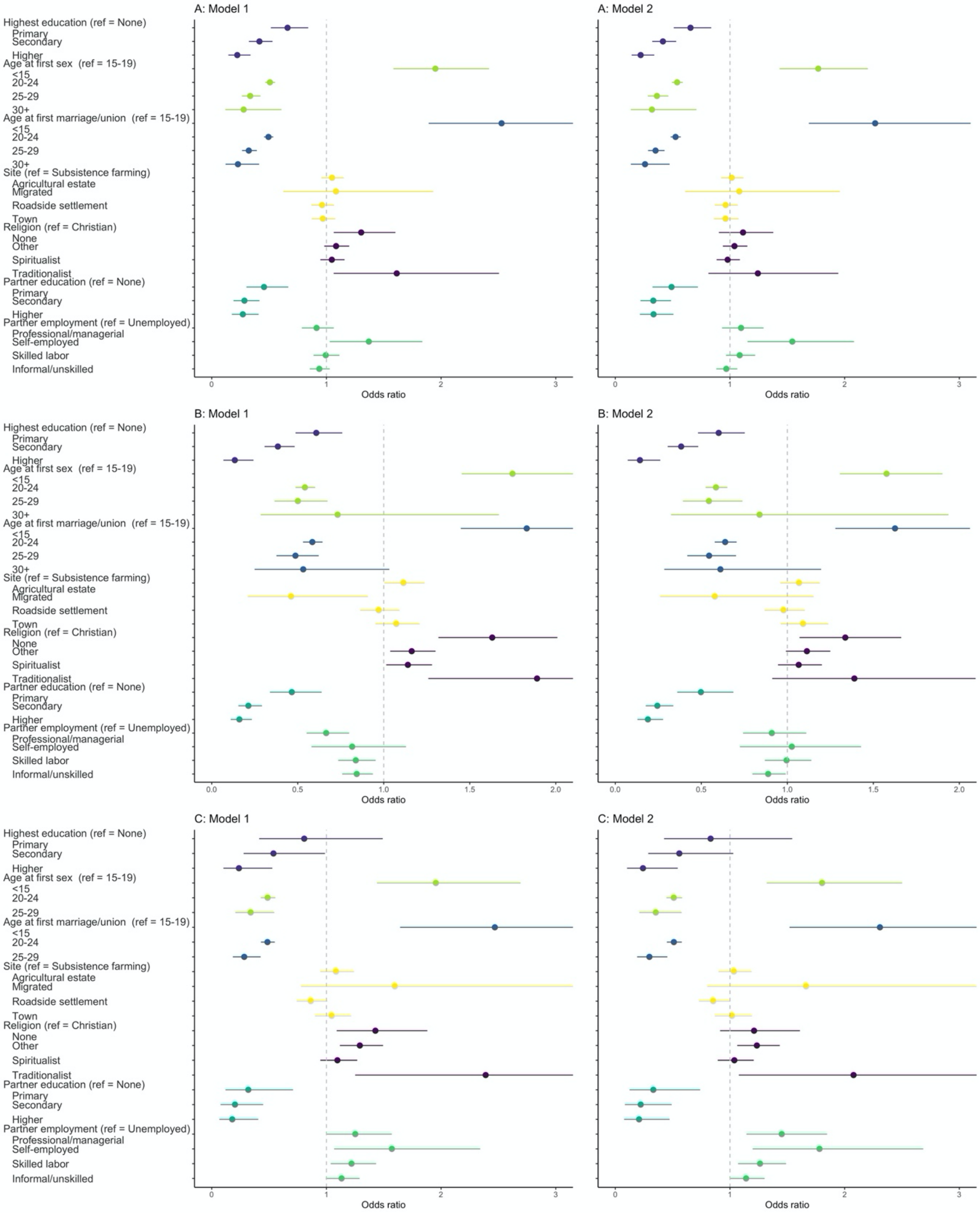

**Results 2. Association between age-disparate relationships and life and relationship outcomes**

Table A2. Association between age-disparate relationships and life and relationship outcomes

| | Odds ratios (95% confidence interval) | | | | | |
|---|---|---|---|---|---|---|
| | All ages, ADR defined as age difference greater than 5 years | | All ages, ADR defined as age difference greater than 10 years | | Ages <=30, ADR defined as age difference greater than 5 years | |
| | model 1 | model 2 | model 1 | model 2 | model 1 | model 2 |
| Employment (ref = Unemployed) | | | | | | |
| Professional/managerial | 0.47 [0.37, 0.61] | 0.62 [0.47, 0.83] | 0.33 [0.22, 0.49] | 0.49 [0.32, 0.75] | 0.63 [0.41, 0.98] | 0.85 [0.53, 1.37] |
| Self-employed | 0.62 [0.35, 1.08] | 0.66 [0.37, 1.18] | 0.32 [0.12, 0.8] | 0.39 [0.15, 0.98] | 0.92 [0.39, 2.19] | 0.88 [0.36, 2.15] |
| Skilled labor | 0.71 [0.55, 0.92] | 0.82 [0.63, 1.07] | 0.84 [0.62, 1.14] | 0.97 [0.71, 1.32] | 0.92 [0.6, 1.43] | 1.06 [0.68, 1.66] |
| Informal/unskilled | 0.97 [0.89, 1.06] | 0.96 [0.87, 1.05] | 1.01 [0.91, 1.11] | 1 [0.91, 1.11] | 1 [0.87, 1.14] | 0.97 [0.85, 1.11] |
| Self-reported health in the last few months (ref = Good health) | | | | | | |
| Recurring sickness | 1.07 [0.96, 1.18] | 1.03 [0.93, 1.14] | 1.19 [1.06, 1.33] | 1.17 [1.04, 1.3] | 1.01 [0.86, 1.2] | 0.97 [0.82, 1.15] |
| Serious illness | 1.31 [1.05, 1.64] | 1.3 [1.03, 1.63] | 1.55 [1.24, 1.95] | 1.58 [1.26, 1.98] | 1.86 [1.28, 2.71] | 1.9 [1.3, 2.78] |
| Partner’s concurrent relationship (ref = None) | | | | | | |
| 2 | 1.47 [1.23, 1.75] | 1.41 [1.17, 1.69] | 2.42 [2.02, 2.91] | 2.36 [1.96, 2.85] | 2.47 [1.68, 3.63] | 2.21 [1.49, 3.26] |
| 3+ | 1.02 [0.66, 1.55] | 0.98 [0.63, 1.51] | 2.31 [1.49, 3.59] | 2.22 [1.41, 3.5] | 2.99 [0.6, 14.96] | 2.06 [0.38, 11.02] |
| Partner bar visit (ref = No) | 1.09 [1.01, 1.18] | 1.09 [1.01, 1.18] | 1.11 [1.02, 1.22] | 1.11 [1.02, 1.22] | 1.2 [1.07, 1.34] | 1.2 [1.07, 1.35] |
| Living arrangement with partner (ref = Living together all times) | | | | | | |
| Living together with occasional trips | 0.86 [0.77, 0.97] | 0.9 [0.8, 1.02] | 0.62 [0.53, 0.71] | 0.63 [0.55, 0.73] | 0.85 [0.73, 1] | 0.89 [0.76, 1.05] |
| Living away for a period (seasonal) | 0.86 [0.73, 1.02] | 0.92 [0.77, 1.09] | 0.77 [0.63, 0.95] | 0.83 [0.67, 1.01] | 1 [0.79, 1.27] | 1.1 [0.86, 1.41] |
| Living apart with regular visits | 0.97 [0.87, 1.08] | 1.02 [0.92, 1.14] | 1 [0.89, 1.12] | 1.03 [0.92, 1.16] | 1.01 [0.87, 1.17] | 1.08 [0.93, 1.25] |
| Living apart | 0.78 [0.62, 0.97] | 0.8 [0.64, 1.01] | 0.73 [0.56, 0.97] | 0.76 [0.57, 1.01] | 0.72 [0.52, 1] | 0.75 [0.54, 1.05] |
| Current marriage status (ref = Still in union) | | | | | | |
| Widowed | 1.32 [1.13, 1.54] | 1.3 [1.12, 1.52] | 1.97 [1.7, 2.29] | 1.97 [1.69, 2.29] | 1.78 [1.12, 2.81] | 1.74 [1.09, 2.78] |

| | | | | | | |
|---|---|---|---|---|---|---|
| Divorced | 1.18 [0.98, 1.43] | 1.23 [1.02, 1.49] | 2.04 [1.69, 2.46] | 2.13 [1.76, 2.58] | 1.39 [1.04, 1.86] | 1.41 [1.05, 1.89] |
| Separated | 0.95 [0.68, 1.33] | 0.93 [0.66, 1.31] | 1.57 [1.1, 2.23] | 1.57 [1.09, 2.27] | 1.02 [0.6, 1.73] | 0.98 [0.58, 1.68] |
| Frequency of contraception use in the last 2-3 years (ref = Most/all of the time) | | | | | | |
| None | 1.07 [0.97, 1.18] | 1.04 [0.94, 1.15] | 1.27 [1.13, 1.43] | 1.25 [1.11, 1.41] | 1.13 [0.92, 1.37] | 1.14 [0.93, 1.39] |
| Some of the time | 1.17 [0.98, 1.39] | 0.62 [0.58, 0.65] | 1.61 [1.33, 1.94] | 1.6 [1.32, 1.93] | 1.02 [0.89, 1.17] | 1.03 [0.9, 1.18] |
| Whether recent pregnancy desired (ref = Yes) | | | | | | |
| Later | 0.78 [0.67, 0.91] | 0.81 [0.69, 0.94] | 0.69 [0.58, 0.82] | 0.7 [0.58, 0.84] | 0.79 [0.65, 0.95] | 0.8 [0.66, 0.97] |
| Not at all | 0.86 [0.78, 0.94] | 0.91 [0.88, 0.95] | 0.86 [0.78, 0.95] | 0.9 [0.81, 1] | 0.95 [0.84, 1.07] | 0.96 [0.85, 1.09] |
| Household wealth index (ref = 1, poorest) | | | | | | |
| 2 | 0.97 [0.86, 1.09] | N/A | 0.89 [0.78, 1.01] | N/A | 0.96 [0.82, 1.13] | N/A |
| 3 | 0.91 [0.79, 1.04] | N/A | 0.95 [0.82, 1.1] | N/A | 0.95 [0.79, 1.14] | N/A |
| 4 | 0.82 [0.7, 0.98] | N/A | 0.69 [0.56, 0.84] | N/A | 0.9 [0.71, 1.14] | N/A |
| 5 (Richest) | 0.66 [0.33, 1.3] | N/A | 0.66 [0.28, 1.53] | N/A | 0.57 [0.21, 1.5] | N/A |

Figure A2. Association between age-disparate relationships and life and relationship outcomes

Panel A: same as presented in the main manuscript (Figure 4), in which ADR is defined as age difference of five or more years.

Panel B: restricting ADR to age difference of ten or more years.

Panel C: restricting to women ages 30 and younger.

Model 1 includes women's age and survey round as covariates

Model 2 includes women's age, survey round, women's highest educational level, and household wealth as covariates

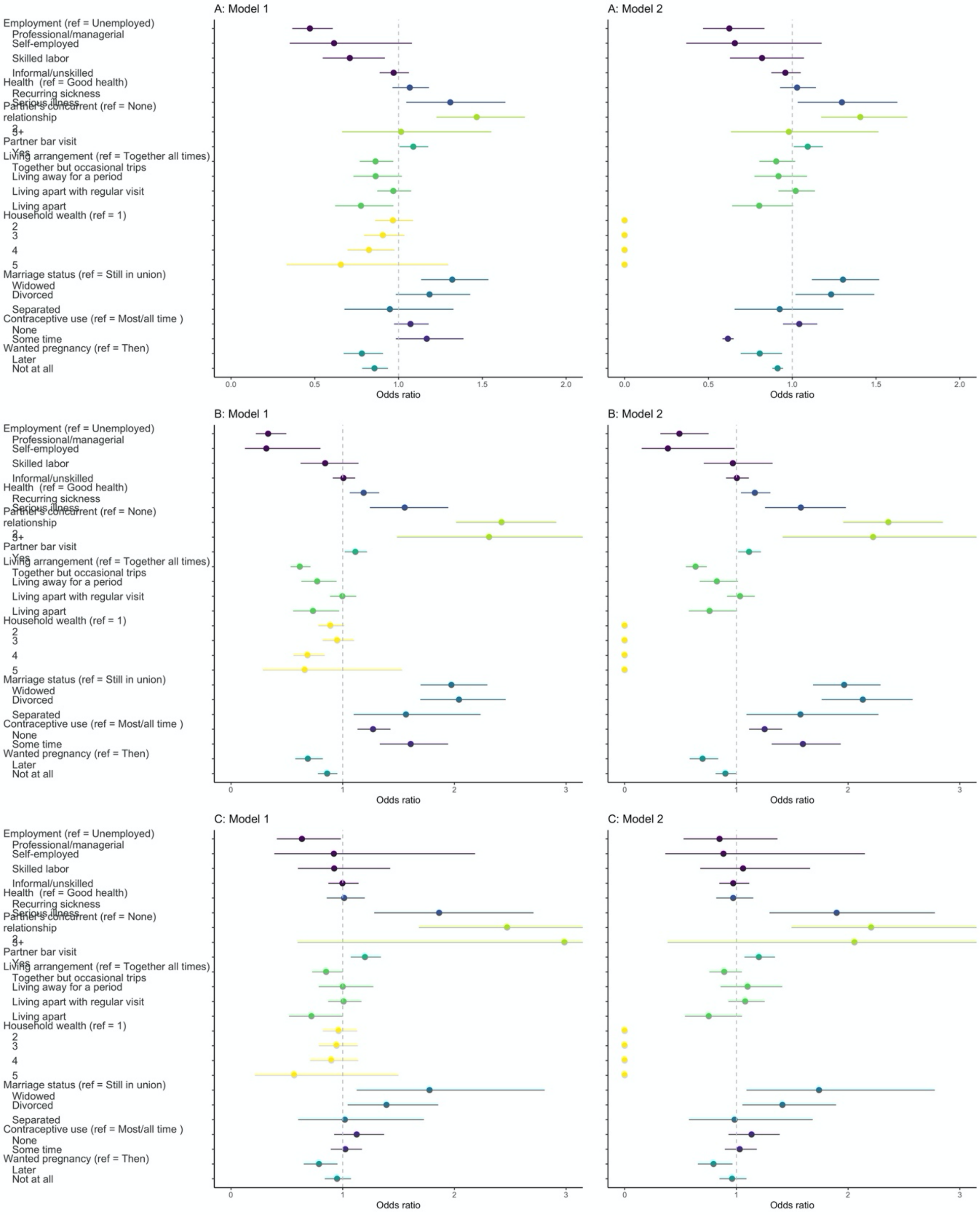

## 2. Sensitivity analyses

We conducted three sets of sensitivity analyses to ensure the robustness of the results presented in the main article. We focused only on the full sample and ADR defined as five or more years of age difference, in comparison to the main results presented in the manuscript (i.e., we did not conduct sensitivity analyses on women younger than age 30 nor ADR defined as ten or more years of age difference).

The three sets of analyses include:

(1) Removing data from survey round 3: we noticed a spike in the proportion of ADR in women with their latest survey round from survey round 3 (Figure 1 in the main text). To ensure the results were not driven primarily by this, we removed all data from this round.

(2) Randomly assigning ADR status to those missing ADR data: There were 4403 (26.4%) of the full sample that we were unable to obtain their ADR status (Table 1 in the main text). They were excluded from the main analysis. For the sensitivity analysis, we randomly assign ADR status to this subset. The estimated ADR prevalence among those with reported ADR status was 58.1%, and we randomly assigned ADR status to the same proportion of women in this subset.

(3) Include only women who are currently still married: in the main analysis, we included all women who were ever married, regardless of their marriage status at the time of their last survey round. To check whether the latest marriage status impacts the reporting of ADR status, we restricted the sample to only those who were still married at the time of the last survey round.

Table A1. Association between determinants and entering age-disparate relationships (ADR)

| | Model 1 (women's age and survey round as covariates) | | | | | | | Model 2 (women's age, survey round, women's highest educational level, and household wealth as covariates) | | | | | | |
|---|---|---|---|---|---|---|---|---|---|---|---|---|---|---|
| Variable | Original result | Sensitivity analysis 1 | (% dif) | Sensitivity analysis 2 | (% dif) | Sensitivity analysis 3 | (% dif) | Original result | Sensitivity analysis 1 | (% dif) | Sensitivity analysis 2 | (% dif) | Sensitivity analysis 3 | (% dif) |
| Highest education (ref = None) | | | | | | | | | | | | | | |
| Primary | 0.66 [0.52-0.84] | 0.67 [0.5-0.88] | 1% | 0.72 [0.68-0.77] | 9% | 0.64 [0.59-0.69] | -4% | 0.66 [0.51-0.84] | 0.65 [0.49-0.86] | -1% | 0.72 [0.68-0.77] | 11% | 0.64 [0.59-0.69] | -3% |
| Secondary | 0.42 [0.32-0.53] | 0.4 [0.3-0.52] | -4% | 0.98 [0.96-1] | 135% | 0.96 [0.93-0.98] | 130% | 0.42 [0.32-0.53] | 0.39 [0.29-0.52] | -6% | 0.98 [0.96-1] | 136% | 0.96 [0.93-0.98] | 131% |
| Higher | 0.22 [0.15-0.34] | 0.24 [0.15-0.38] | 10% | 0.52 [0.45-0.62] | 136% | 0.43 [0.36-0.53] | 95% | 0.22 [0.14-0.34] | 0.24 [0.15-0.39] | 10% | 0.52 [0.44-0.61] | 134% | 0.42 [0.35-0.52] | 91% |
| Age at first sex (ref = 15-19) | | | | | | | | | | | | | | |
| < 15 | 1.95 [1.58-2.42] | 1.8 [1.43-2.29] | -8% | 1.61 [1.36-1.9] | -18% | 1.98 [1.58-2.51] | 2% | 1.77 [1.43-2.2] | 1.61 [1.27-2.05] | -9% | 1.52 [1.28-1.8] | -14% | 1.82 [1.44-2.31] | 3% |
| 20-24 | 0.51 [0.47-0.55] | 0.49 [0.44-0.54] | -4% | 0.63 [0.59-0.68] | 24% | 0.5 [0.46-0.55] | -2% | 0.54 [0.49-0.59] | 0.52 [0.47-0.57] | -3% | 0.66 [0.61-0.71] | 22% | 0.53 [0.48-0.58] | -3% |
| 25-29 | 0.34 [0.26-0.43] | 0.3 [0.23-0.39] | -10% | 0.43 [0.36-0.53] | 29% | 0.33 [0.25-0.42] | -2% | 0.36 [0.28-0.46] | 0.33 [0.25-0.43] | -9% | 0.46 [0.38-0.56] | 27% | 0.35 [0.27-0.46] | -2% |
| 30+ | 0.28 [0.12-0.61] | 0.29 [0.12-0.67] | 6% | 0.36 [0.19-0.67] | 30% | 0.32 [0.14-0.72] | 16% | 0.32 [0.14-0.71] | 0.34 [0.14-0.8] | 8% | 0.4 [0.21-0.75] | 26% | 0.37 [0.16-0.84] | 16% |
| Age at first marriage (ref = 15-19) | | | | | | | | | | | | | | |
| < 15 | 2.53 [1.89-3.44] | 2.27 [1.65-3.19] | -10% | 1.64 [1.33-2.04] | -35% | 2.63 [1.92-3.68] | 4% | 2.27 [1.69-3.1] | 2.01 [1.45-2.84] | -11% | 1.57 [1.26-1.95] | -31% | 2.37 [1.72-3.34] | 5% |
| 20-24 | 0.49 [0.46-0.54] | 0.48 [0.44-0.53] | -3% | 0.61 [0.57-0.66] | 24% | 0.49 [0.45-0.54] | 0% | 0.53 [0.48-0.57] | 0.51 [0.47-0.56] | -2% | 0.64 [0.6-0.69] | 22% | 0.52 [0.47-0.57] | -1% |
| 25-29 | 0.32 [0.26-0.39] | 0.31 [0.25-0.39] | -5% | 0.44 [0.38-0.52] | 37% | 0.32 [0.26-0.4] | -1% | 0.35 [0.29-0.43] | 0.34 [0.27-0.43] | -3% | 0.47 [0.4-0.55] | 34% | 0.35 [0.28-0.43] | -1% |
| 30+ | 0.23 [0.12-0.41] | 0.25 [0.12-0.47] | 8% | 0.44 [0.29-0.67] | 92% | 0.26 [0.14-0.47] | 13% | 0.26 [0.14-0.47] | 0.29 [0.14-0.55] | 11% | 0.48 [0.31-0.74] | 85% | 0.29 [0.15-0.54] | 13% |
| Site (ref = Subsistence farming) | | | | | | | | | | | | | | |
| Agricultural estate | 1.05 [0.96-1.15] | 1.01 [0.91-1.12] | -4% | 1.01 [0.93-1.09] | -4% | 1.05 [0.95-1.15] | 0% | 1.02 [0.92-1.12] | 0.97 [0.88-1.08] | -4% | 0.98 [0.9-1.06] | -4% | 1.01 [0.91-1.12] | -1% |
| Migrated | 1.08 [0.62-1.93] | 1.3 [0.75-2.32] | 20% | 1.07 [0.65-1.8] | -1% | 1.25 [0.67-2.42] | 15% | 1.08 [0.61-1.96] | 1.29 [0.73-2.34] | 19% | 1.05 [0.63-1.79] | -3% | 1.26 [0.67-2.48] | 17% |
| Roadside settlement | 0.96 [0.87-1.07] | 0.92 [0.82-1.03] | -4% | 0.94 [0.86-1.03] | -3% | 0.92 [0.82-1.02] | -5% | 0.96 [0.87-1.07] | 0.92 [0.82-1.03] | -5% | 0.94 [0.86-1.03] | -2% | 0.91 [0.81-1.02] | -6% |
| Town | 0.97 [0.87-1.08] | 0.94 [0.84-1.06] | -3% | 0.94 [0.86-1.02] | -3% | 0.96 [0.86-1.07] | -1% | 0.96 [0.86-1.08] | 0.94 [0.83-1.06] | -2% | 0.93 [0.84-1.02] | -4% | 0.94 [0.84-1.06] | -2% |
| Religion (ref = Christian) | | | | | | | | | | | | | | |
| None | 1.3 [1.06-1.6] | 1.22 [0.97-1.55] | -6%* | 1.19 [1.01-1.41] | -8% | 1.36 [1.1-1.7] | 5% | 1.11 [0.9-1.38] | 1.03 [0.81-1.31] | -8% | 1.06 [0.9-1.26] | -5% | 1.18 [0.94-1.49] | 6% |
| Other | 1.08 [0.98-1.2] | 1.12 [1-1.25] | 3% | 1.07 [0.98-1.16] | -1% | 1.1 [0.99-1.23] | 2% | 1.04 [0.94-1.15] | 1.06 [0.95-1.19] | 2% | 1.04 [0.95-1.13] | 0% | 1.05 [0.94-1.18] | 1% |
| Spiritualist | 1.05 [0.95-1.16] | 1.08 [0.97-1.2] | 3% | 1.06 [0.97-1.15] | 1% | 1.04 [0.94-1.16] | 0% | 0.98 [0.88-1.09] | 1 [0.9-1.12] | 2% | 1 [0.92-1.1] | 2% | 0.99 [0.88-1.1] | 1% |
| Traditionalist | 1.61 [1.06-2.5] | 2.26 [1.35-3.93] | 40% | 1.28 [0.94-1.77] | -21% | 1.69 [1.09-2.68] | 5% | 1.24 [0.81-1.94] | 1.73 [1.03-3.03] | 39%* | 1.09 [0.8-1.52] | -12% | 1.33 [0.85-2.13] | 7% |
| Partner education (ref = None) | | | | | | | | | | | | | | |
| Primary | 0.46 [0.3-0.67] | 0.34 [0.2-0.55] | -26% | 0.53 [0.37-0.75] | 16% | 0.44 [0.29-0.66] | -3% | 0.49 [0.32-0.72] | 0.38 [0.22-0.62] | -22% | 0.56 [0.39-0.8] | 15% | 0.48 [0.31-0.71] | -3% |
| Secondary | 0.29 [0.19-0.42] | 0.22 [0.13-0.36] | -23% | 0.34 [0.24-0.48] | 19% | 0.28 [0.18-0.41] | -3% | 0.33 [0.22-0.49] | 0.28 [0.17-0.46] | -15% | 0.39 [0.27-0.55] | 17% | 0.32 [0.21-0.48] | -3% |
| Higher | 0.27 [0.18-0.41] | 0.25 [0.14-0.41] | -10% | 0.33 [0.22-0.48] | 20% | 0.27 [0.17-0.42] | 0% | 0.33 [0.21-0.51] | 0.33 [0.19-0.55] | -2% | 0.4 [0.27-0.58] | 19% | 0.33 [0.21-0.52] | 0% |
| Partner employment (ref = Unemployed) | | | | | | | | | | | | | | |
| Professional/managerial | 0.91 [0.79-1.06] | 0.88 [0.75-1.04] | -3% | 0.93 [0.8-1.08] | 1% | 0.89 [0.76-1.03] | -3% | 1.1 [0.93-1.29] | 1 [0.84-1.2] | -9% | 1.09 [0.93-1.28] | -1% | 1.04 [0.88-1.23] | -5% |
| Self-employed | 1.37 [1.03-1.84] | 1.36 [1-1.85] | -1% | 1.38 [1.04-1.85] | 1% | 1.35 [1.01-1.82] | -1% | 1.54 [1.15-2.08] | 1.49 [1.1-2.04] | -4% | 1.55 [1.16-2.08] | 0% | 1.51 [1.12-2.04] | -2% |
| Skilled labor | 0.99 [0.89-1.11] | 0.96 [0.85-1.09] | -4% | 0.99 [0.89-1.11] | 0% | 0.98 [0.87-1.1] | -2% | 1.09 [0.97-1.22] | 1.03 [0.9-1.17] | -5% | 1.07 [0.96-1.21] | -1% | 1.06 [0.94-1.19] | -2% |
| Informal/unskilled | 0.94 [0.86-1.03] | 0.95 [0.85-1.05] | 1% | 0.94 [0.85-1.03] | 0% | 0.93 [0.84-1.02] | -1% | 0.97 [0.88-1.06] | 0.97 [0.87-1.08] | 0% | 0.96 [0.88-1.05] | -1% | 0.95 [0.86-1.05] | -2% |

* change in statistical significance

** change in statistical significance and direction (i.e., OR > 1 → OR < 1, or OR <1 → OR > 1)

Table A2. Association between age-disparate relationships (ADR) and life and relationship outcomes

| | Model 1 (women's age and survey round as covariates) | | | | | | | Model 2 (women's age, survey round, women's highest educational level, and household wealth as covariates) | | | | | | |
|---|---|---|---|---|---|---|---|---|---|---|---|---|---|---|
| Variable | Original result | Sensitivity analysis 1 | (% dif) | Sensitivity analysis 2 | (% dif) | Sensitivity analysis 3 | (% dif) | Original result | Sensitivity analysis 1 | (% dif) | Sensitivity analysis 2 | (% dif) | Sensitivity analysis 3 | (% dif) |
| Employment (ref = Unemployed) | | | | | | | | | | | | | | |
| Professional/ managerial | 0.47 [0.37-0.61] | 0.48 [0.36-0.63] | 2% | 0.52 [0.42-0.66] | 11% | 0.52 [0.4-0.68] | 10% | 0.62 [0.47-0.83] | 0.61 [0.45-0.84] | -2% | 0.65 [0.5-0.83] | 3% | 0.68 [0.5-0.91] | 8% |
| Self-employed | 0.62 [0.35-1.08] | 0.76 [0.42-1.37] | 23% | 0.61 [0.37-1] | -1% | 0.74 [0.41-1.35] | 21% | 0.66 [0.37-1.18] | 0.82 [0.44-1.51] | 24% | 0.64 [0.39-1.06] | -2% | 0.79 [0.43-1.47] | 21% |
| Skilled labor | 0.71 [0.55-0.92] | 0.71 [0.52-0.96] | 0% | 0.72 [0.59-0.89] | 2% | 0.69 [0.52-0.92] | -2% | 0.82 [0.63-1.07] | 0.8 [0.58-1.09] | -3% | 0.8 [0.65-0.99] | -2%* | 0.79 [0.59-1.07] | -3% |
| Informal/ unskilled | 0.97 [0.89-1.06] | 0.93 [0.84-1.04] | -4% | 0.95 [0.89-1.03] | -2% | 0.93 [0.84-1.02] | -4% | 0.96 [0.87-1.05] | 0.93 [0.83-1.03] | -3% | 0.94 [0.87-1.01] | -2% | 0.91 [0.83-1.01] | -5% |
| Health (ref = Good health) | | | | | | | | | | | | | | |
| Recurring sickness | 1.07 [0.96-1.18] | 1.03 [0.92-1.16] | -3% | 1.03 [0.94-1.12] | -4% | 1.01 [0.9-1.13] | -5% | 1.03 [0.93-1.14] | 1 [0.89-1.12] | -3% | 1 [0.91-1.09] | -3% | 0.98 [0.87-1.1] | -5% |
| Serious illness | 1.31 [1.05-1.64] | 0.93 [0.68-1.26] | -29%** | 1.18 [0.99-1.41] | -10%* | 1.24 [0.95-1.61] | -5%* | 1.3 [1.03-1.63] | 0.89 [0.65-1.22] | -31%** | 1.16 [0.97-1.39] | -10%* | 1.26 [0.97-1.65] | -3%* |
| Partner's concurrent relationship (ref = None) | | | | | | | | | | | | | | |
| 2 | 1.47 [1.23-1.75] | 1.47 [1.23-1.75] | 0% | 1.31 [1.12-1.54] | -11% | 1.54 [1.27-1.87] | 5% | 1.41 [1.17-1.69] | 1.41 [1.17-1.69] | 0% | 1.27 [1.08-1.49] | -10% | 1.46 [1.2-1.78] | 4% |
| 3+ | 1.02 [0.66-1.55] | 1.02 [0.66-1.55] | 0% | 1.1 [0.75-1.62] | 9% | 1.01 [0.63-1.62] | -1% | 0.98 [0.63-1.51] | 0.98 [0.63-1.52] | 0% | 1.06 [0.72-1.57] | 8% | 0.95 [0.59-1.54] | -3% |
| Partner bar visit | | | | | | | | | | | | | | |
| Yes | 1.09 [1.01-1.18] | 1.03 [0.95-1.13] | -5%* | 1.09 [1.01-1.18] | 0% | 1.08 [1-1.17] | -1%* | 1.09 [1.01-1.18] | 1.05 [0.96-1.14] | -4%* | 1.09 [1.01-1.18] | 0% | 1.09 [1-1.18] | 0% |
| Living arrangement (ref = Together all times) | | | | | | | | | | | | | | |
| Together but occasional trips | 0.86 [0.77-0.97] | 0.92 [0.81-1.04] | 6%* | 0.86 [0.77-0.96] | 0% | 0.86 [0.77-0.97] | 0% | 0.9 [0.8-1.02] | 0.98 [0.86-1.11] | 8% | 0.9 [0.8-1.01] | 0% | 0.91 [0.8-1.02] | 0% |
| Living away for a period | 0.86 [0.73-1.02] | 0.87 [0.73-1.05] | 1% | 0.87 [0.74-1.03] | 1% | 0.86 [0.72-1.02] | -1% | 0.92 [0.77-1.09] | 0.95 [0.78-1.14] | 3% | 0.93 [0.78-1.1] | 1% | 0.92 [0.77-1.09] | 0% |
| Living apart with regular visit | 0.97 [0.87-1.08] | 0.89 [0.78-1] | -9%* | 0.96 [0.87-1.07] | -1% | 0.95 [0.85-1.05] | -2% | 1.02 [0.92-1.14] | 0.95 [0.84-1.08] | -7% | 1.01 [0.91-1.13] | -1% | 0.99 [0.89-1.11] | -3% |
| Living apart | 0.78 [0.62-0.97] | 0.81 [0.63-1.02] | 4%* | 0.77 [0.62-0.96] | 0% | 0.78 [0.63-0.98] | 1% | 0.8 [0.64-1.01] | 0.85 [0.67-1.08] | 6% | 0.79 [0.63-0.99] | -1%* | 0.81 [0.65-1.02] | 1% |
| Marriage status (ref = Still in union) | | | | | | | | | | | | | | |
| Widowed | 1.32 [1.13-1.54] | 1.24 [1.03-1.49] | -6% | 1.11 [1.01-1.23] | -16% | N/A | N/A | 1.3 [1.12-1.52] | 1.21 [1.01-1.46] | -7% | 1.1 [1-1.22] | -15%* | N/A | N/A |
| Divorced | 1.18 [0.98-1.43] | 1.13 [0.86-1.5] | -5% | 1.11 [1-1.23] | -7% | N/A | N/A | 1.23 [1.02-1.49] | 1.17 [0.89-1.56] | -5%* | 1.12 [1-1.24] | -9% | N/A | N/A |
| Separated | 0.95 [0.68-1.33] | 0.76 [0.48-1.2] | -20% | 0.94 [0.8-1.12] | -1% | N/A | N/A | 0.93 [0.66-1.31] | 0.78 [0.49-1.24] | -16% | 0.93 [0.78-1.11] | 1% | N/A | N/A |
| Contraceptive use (ref = Most/all time) | | | | | | | | | | | | | | |
| None | 1.07 [0.97-1.18] | 1.04 [0.94-1.15] | -3% | 1.07 [0.98-1.16] | -1% | 1.06 [0.96-1.17] | -1% | 1.04 [0.94-1.15] | 1 [0.9-1.11] | -4% | 1.04 [0.95-1.14] | 0% | 1.04 [0.94-1.15] | 0% |
| Some time | 1.17 [0.98-1.39] | 1.05 [0.86-1.29] | -10% | 1.12 [0.97-1.3] | -4% | 1.15 [0.96-1.37] | -2% | 0.62 [0.58-0.65] | 1.04 [0.85-1.29] | 70%** | 0.62 [0.59-0.65] | 1% | 0.63 [0.6-0.67] | 2% |
| Wanted pregnancy (ref = Then) | | | | | | | | | | | | | | |
| Later | 0.78 [0.67-0.91] | 0.86 [0.73-1.02] | 11%* | 0.84 [0.74-0.96] | 8% | 0.8 [0.68-0.93] | 2% | 0.81 [0.69-0.94] | 0.9 [0.76-1.07] | 12%* | 0.86 [0.75-0.98] | 7% | 0.82 [0.7-0.96] | 2% |
| Not at all | 0.86 [0.78-0.94] | 0.86 [0.78-0.96] | 1% | 0.89 [0.82-0.96] | 3% | 0.87 [0.79-0.96] | 2% | 0.91 [0.88-0.95] | 0.9 [0.81-1] | -1%* | 0.9 [0.87-0.93] | -2% | 0.86 [0.83-0.9] | -5% |
| Household wealth (ref = 1) | | | | | | | | | | | | | | |
| 2 | 0.97 [0.86-1.09] | 0.99 [0.86-1.12] | 2% | 0.98 [0.89-1.08] | 1% | 0.99 [0.88-1.12] | 3% | N/A | N/A | N/A | N/A | N/A | N/A | N/A |
| 3 | 0.91 [0.79-1.04] | 0.9 [0.78-1.04] | -1% | 0.95 [0.85-1.06] | 5% | 0.96 [0.83-1.1] | 6% | N/A | N/A | N/A | N/A | N/A | N/A | N/A |
| 4 | 0.82 [0.7-0.98] | 0.83 [0.69-1] | 1%* | 0.82 [0.71-0.95] | 0% | 0.88 [0.74-1.05] | 7%* | N/A | N/A | N/A | N/A | N/A | N/A | N/A |
| 5 | 0.66 [0.33-1.3] | 0.74 [0.35-1.58] | 13% | 0.67 [0.37-1.23] | 3% | 0.84 [0.41-1.73] | 29% | N/A | N/A | N/A | N/A | N/A | N/A | N/A |

* change in statistical significance
** change in statistical significance and direction (i.e., OR > 1 → OR < 1, or OR <1 → OR > 1)
